# Mannose-binding lectin protein and its association to clinical outcomes in COPD: a longitudinal study

**DOI:** 10.1186/s12931-015-0306-3

**Published:** 2015-12-18

**Authors:** Jyotshna Mandal, Bijaya Malla, Rudi Steffensen, Luigi Costa, Adrian Egli, Marten Trendelenburg, Francesco Blasi, Kostantinos Kostikas, Tobias Welte, Antoni Torres, Renaud Louis, Wim Boersma, Branislava Milenkovic, Joachim Aerts, Gernot G. U. Rohde, Alicia Lacoma, Katharina Rentsch, Michael Roth, Michael Tamm, Daiana Stolz

**Affiliations:** Clinic of Pulmonary Medicine and Respiratory Cell Research, University Hospital Basel, Petersgraben, 44031 Basel, Switzerland; Department of Clinical Immunology, Aalborg University Hospital, Aalborg, Denmark; Infection Disease Department, University Hospital Basel, Basel, Switzerland; Department of Biomedicine and Division of Internal Medicine, University Hospital Basel, Basel, Switzerland; Department of Pathophysiology and Transplantation, Università degli Studi di Milano, IRCCS Fondazione Cà Granda Policlinico, Milan, Italy; Thessaly Medical School, Thessaloniki, Greece; Medical School Hannover, Hannover, Germany; Hospital Clinic, Barcelona, Spain; University of Liege, Liege, Belgium; Medisch Centrum Alkmaar, Alkmaar, Netherlands; Faculty of Medicine, University of Belgrade; Clinic for Pulmonary Diseases, Belgrade, Serbia; Amphia Hospital, Breda, Netherlands; Maastricht University Medical Center, Maastricht, The Netherlands; Department of Microbiology, Hospital Universitari Germans Trias i Pujol, CIBER Enfermedades Respiratorias, Badalona, Spain; Department of Laboratorial Medicine, University Hospital Basel, Basel, Switzerland

**Keywords:** Lung function, Haplotypes, Exacerbation, Survival rate

## Abstract

**Background:**

Functional deficiency of mannose-binding lectin (MBL) may contribute to the pathogenesis of chronic obstructive pulmonary disease. We hypothesized that specific MBL2 gene polymorphisms and circulating MBL protein levels are associated with clinically relevant outcomes in the Predicting Outcome using systemic Markers In Severe Exacerbations of COPD PROMISE-COPD cohort.

**Methods:**

We followed 277 patients with stable COPD GOLD stage II-IV COPD over a median period of 733 days (IQR 641–767) taking survival as the primary outcome parameter. Patients were dichotomized as frequent (≥2 AECOPD/year) or infrequent exacerbators. Serum MBL levels and single nucleotide polymorphisms of the MBL2 gene were assessed at baseline.

**Results:**

The *MBL2-HYPD* haplotype was significantly more prevalent in frequent exacerbators (OR: 3.33; 95 % CI, 1.24–7.14, *p* = 0.01). The median serum MBL concentration was similar in frequent (607 ng/ml, [IQR; 363.0–896.0 ng/ml]) and infrequent exacerbators (615 ng/ml, [IQR; 371.0–942.0 ng/ml]). Serum MBL was not associated with lung function characteristics or bacterial colonization in sputum. However, high serum MBL at stable state was associated with better survival compared to low MBL (*p* = 0.046, log rank test).

**Conclusions:**

In COPD, the *HYPD* haplotype of *MBL2* gene is associated with frequent exacerbations and high serum MBL is linked to increased survival.

The PROMISE-COPD study was registered at www.controlled-trials.com under the identifier ISRCTN99586989.

**Electronic supplementary material:**

The online version of this article (doi:10.1186/s12931-015-0306-3) contains supplementary material, which is available to authorized users.

## Background

The World Health Organization projects that Chronic Obstructive Pulmonary Disease (COPD) will become the third leading cause of death worldwide by 2030 [1]. The increasing COPD mortality might be associated with the aging population, tobacco use, air pollution, and respiratory infection [2]. In COPD patients, exacerbation causes a mounting economic burden by frequent hospitalization and by chronic disability. Recurrent exacerbations are associated with accelerated lung function deterioration, reduction of quality of life, and decreased survival in COPD. Some COPD patients are susceptible to exacerbations requiring frequent hospital visits and hospitalizations. Thus, frequent exacerbation has been recognised as a major clinically relevant COPD phenotype [3]. The identification of predictive markers for these episodic and recurrent worsening of symptoms may help to improve the management of COPD [4].

Mannose binding lectin (MBL) is an important factor in the innate immune system that binds to carbohydrate regions of bacterial cell walls and viral capsules. Binding of MBL protein activates the complement system via MBL-associated serine proteases, eventually leading to the clearance of pathogens [5]. Polymorphisms of the human *MBL2* gene are associated with deficiency of serum MBL protein levels that may cause susceptibility to infection [4]. The polymorphisms that control the serum MBL protein levels are located in the promoter region, and in exon 1 of the human *MBL2* gene [6]. In general, carriers with different polymorphisms in exon 1 present low MBL serum levels. Not only that *MBL2* polymorphisms and serum MBL deficiency have been found to be associated with susceptibility to bacterial and viral diseases, they have also been linked to non-communicable diseases such as cystic fibrosis, and COPD [7, 8]. Serum MBL levels for genotypes *XA/O* and *O/O* had a mean serum level below 100 ng/ml [4]. Furthermore, COPD patients with the *“O”-*allele are reported to suffer from recurrent infective exacerbations. Thus, we hypothesized that specific *MBL2* polymorphisms and circulating MBL levels are associated with clinically relevant outcomes in COPD. To prove our hypothesis, we evaluated serum MBL levels and polymorphisms in the *MBL2* gene in a well-characterized cohort of COPD patients.

## Methods

### Study population

This nested cohort study included 277 patients enrolled in the investigator initiated PROMISE-COPD study (Predicting Outcome using systemic Markers In Severe Exacerbations of COPD). The Patients were recruited at a baseline visit after consent from 11 primary and tertiary study centres across Europe during 2008–2011. The study was approved by the Ethics Committee Beider Basel (EKBB 295/07), and was registered at www.controlled-trials.com (ISRCTN99586989). The patients had to be >40 years, smoking history of ≥ 10 pack years, and be at a stable state defined as clinical stability for 4 weeks after resolution of the last exacerbation. Patients with pulmonary conditions other than COPD (asthma, bronchiectasis, cystic fibrosis), immunosuppressive diseases, chronic steroid use >10 mg/day prednisolone-equivalent, musculo-skeletal process preventing ambulation, and estimated life expectancy <6 months were excluded.

### Baseline and scheduled visits assessment

COPD exacerbation was defined as an acute change from baseline in dyspnea, cough, and/or sputum production beyond normal day-to-day variation that necessitates use of antibiotics, glucocorticoids, or both [9]. Spirometry was performed following American Thoracic Society guidelines. Patients were categorized into COPD stages (II-IV); (post-bronchodilator FEV1/FVC < 70 %; FEV1 < 80 % predicted) and grouped according to the GOLD 2011/ 2013 classification [2, 10]. Patients were grouped into infrequent exacerbators (0–1 exacerbation/year) and frequent exacerbators (≥2 exacerbations/year). Outcome (all-cause mortality) was evaluated by contacting the patients, family physicians, or by checking public registries. The median follow-up time of the study was 733 days (IQR; 641–767 days). Patients answered the St. George’s Respiratory Questionnaire (SGRQ) and the Short Form Health Survey (SF-36) at the time of enrolment. Spontaneously expectorated sputum samples were obtained and were examined by using standard microbiology culture techniques. Sputum samples were examined for Gram staining. Good sputum quality was defined as <25 epithelial cells in 100× augmentation [11]. Serum samples for MBL measurements and genotyping were collected at stable state.

### Genotyping of *MBL2* gene

Three single nucleotide polymorphisms (SNPs) within the *MBL2* promoter (*H/L*; −550, rs11003125), (*X/Y*; −221, rs7096206), (*P/Q*; +4, rs7095891) and three SNPs in *MBL2* exon 1 (*A/D*; codon52, rs5030737), *(A/B*; codon54, rs1800450), (*A/C*; codon57, rs1800451) were determined by real-time polymerase chain reaction (RT-PCR) using minor-groove-binder (MGB) *TaqMan* probes (Applied Biosystems, Foster City, CA, USA). Genotyping was achieved by end-point fluorescence using SDS software (version 2.3) [12, 13]. The SNPs used to characterise the *MBL2* alleles are listed in Table 1.

**Table 1:** Mannose-binding lectin 2 (*MBL2*) gene polymorphisms in patients with infrequent and frequent exacerbation

Single nucleotide polymorphisms	Infrequent exacerbation n (%)	Frequent exacerbation n (%)	*p* value
Promoter region			
HL (−550 G > C; *rs*11003125)			0.03
C/C (L/L)	77 (50.0)	46 (37.4)	
G/C (H/L)	57 (37.0)	48 (39.0)	
G/G (H/H)	20 (13.0)	29 (23.6)	
YX (−221 G > C; *rs*7096206)			0.64
G/G (Y/Y)	92 (59.7)	80 (65.0)	
G/C (Y/X)	53 (34.4)	36 (29.3)	
C/C (X/X)	9 (5.8)	7 (5.7)	
Exon 1			
PQ (+4 C > T; *rs*7095891)			0.65
C/C (P/P)	89 (57.8)	77 (62.6)	
C/T (P/Q)	47 (30.5)	35 (28.5)	
T/T (Q/Q)	18 (11.7)	11 (8.9)	
A/D (+223 C > T; *rs*5030737)			0.03
A/A (C/C)	124 (91.9)	119 (83.8)	
A/D (C/T)	12 (7.8)	21 (17.1)	
D/D (T/T)	0 (0)	1 (0.8)	
A/B (+230 G > A; *rs*1800450)			
A/A (G/G)	129 (83.8)	106(86.2)	0.79
A/B (G/A)	23 (14.9)	15 (12.2)	
B/B (A/A)	2 (1.3)	2 (1.6)	
A/C (+239 G > A; *rs*1800451)			
A/A (G/G)	146 (94.8)	119 (96.7)	0.27
A/C (G/C)	8 (5.2)	3 (2.4)	
C/C (A/A)	0 (0)	1 (0.8)	

The distribution of each polymorphism in promoter and exon 1 region of *MBL2* is shown in infrequent and frequent exacerbation. The *HL* and *AD* polymorphisms were significantly different between exacerbation groups. The *rs* number for each SNP represents the reference Single nucleotide polymorphisms identification number

### Measurement of serum MBL concentration

Serum MBL was measured in duplicate by enzyme-linked immunosorbent assay (R&D Systems, Inc., Minneapolis, MN, USA) following the manufacturer protocol. The serum was assigned as “low” for MBL levels below the 75^th^ quartile (<934 ng/ml), and as “high” above the 75^th^ percentile (≥934 ng/ml) [14, 15].

### Statistical analysis

Statistical analysis was done by SPSS (Version 21, IBM Corp., NY, USA), and *p*-values less than 0.05 were considered significant. Baseline data are presented as mean or median values (continuous variables), while discrete data are as percentages. The association of *MBL2* polymorphisms and low and high serum MBL, with exacerbation was tested by Chi-square test. The Metric data was analysed by Students-t-test or Mann–Whitney-U test. The Kaplan-Meier log rank test was used to test the null hypothesis: “no difference in time to death”.

## Results

### Study population

Clinical characteristics of patients are summarized in Table 2, showing a mean age of 68 years with 70 % being male. The majority of patients were ex-smokers with a median cigarette consumption of 40 pack-years. The median duration of COPD was 60 months and 185 (66.8 %) patients had exacerbations ranging from 1–15 physician visits in previous years. Of those patients, 35.9 % had a history of hospitalization in the previous year, 9 % of which required intensive care unit stay. GOLD classification showed that 50 % of the patients had moderate COPD (GOLD II; 46.6 %). 123 (44.4 %) patients were classified as frequent exacerbators.

**Table 2:** Demographic characteristics of study population

Total *n* = 277	
Patient Characteristics	Values^a£^
Age (years)	67.8 ± 9.5
Sex (Male)	190 (68.6)
Race (Caucasian)	274 (98.9)
BMI (kg/m^2^)	26.3 (23.3–29.4)
Smoking status	
Current smokers, (n) %	74 (26.7)
Median Pack-years, median, IQR	50 (35–80)
Comorbidities	
Congestive heart disease, %	17.5
Diabetes mellitus, %	12.9
Liver disease, %	2.9
Malignant solid tumour, %	4.0
Arterial hypertension, %	56.4
Pulmonary arterial hypertension, %	8.0
History of exacerbations in COPD patients in previous years	
Median duration of COPD (months), median IQR	84 (44.5–130)
Duration of physician-diagnosed COPD (months), median, IQR	60 (29–120)
Required unscheduled urgent physician visit, median, IQR	1 (0–2)
Required hospitalization, median, IQR	0 (0–1)
Antibiotics use, median, IQR	1 (0–1)
Current medication for COPD	
SABA + SAMA, %	20.6
LABA + ICS, %	73.5
Single drug inhaler	
SABA, %	31.4
LABA, %	12.27
SAMA, %	6.2
LAMA, %	71.7
ICS, %	11.7
Lung function tests	
FEV1,L post-BD, mean ± S.D.	1.4 ± 0.6
FEV1 % predicted post-BD, mean ± S.D.	48.2 ± 17.5
FVC,L post-BD, mean ± S.D.	2.9 ± 3.8
FVC % predicted post-BD, mean ± S.D.	77.9 ± 23.2
FEV1/FVC post-BD, mean ± S.D.	48.0 ± 13.6
GOLD Stages	
GOLD II, (n) %	129 (47.3)
GOLD III, (n) %	104 (38.1)
GOLD IV, (n) %	40 (14.6)
SGRQ - Quality of life	
Symptoms score, median IQR	46.3 (29.3–65.8)
Activity score, median IQR	59.4 (42.4–79.2)
Impact score, median IQR	30.0 (18.3–46.5)
Total score, median IQR	41.8 (29.5–57.8)
SF-36 Health Survey	
Physical Function, median IQR	50.0 (25.0–70.0)
Role Function, median IQR	50.0 (0.0–100.0)
Role Emotional, median IQR	100.0 (8.3–100.0)
Social Function, median IQR	75.0 (50.0–100.0)
Mental Health, median IQR	65.0 (55.0–80.0)
Body Pain, median IQR	74.0 (51.0–100.0)
Vitality, median IQR	50.0 (37.5–62.5)
General Health, median IQR	45.0 (28.3–65.0)
Exercise performance	
6MWD, m, mean ± S.D.	375.5 ± 102.6
Peripheral Oxygen saturation at rest, mean ± S.D.	94.5 ± 2.7
Lowest oxygen saturation during test, mean ± S.D.	89.6 ± 6.0
Heart rate at rest (beats/min), mean ± S.D.	80.8 ± 14.7
Highest heart rate during test (beats/min), mean ± S.D.	105.9 ± 19.49
6 min’ walk BORG score, median IQR	4 (3–6)
BODE index, median IQR	3 (1–4)
Serum MBL level	
Serum MBL concentration, ng/ml, median, IQR	612 (365.5–933.0)
Low serum MBL level (<75th quartile; <934 ng/ml), (n) %	208 (75.1)
High serum MBL level (≥75th percentile; ≥934 ng/ml), (n) %	69 (24.9)

^a^The continuous data are presented as mean ± SD or median (IQR). ^£^The count data is presented as No. (%). *GOLD* Global initiative for chronic obstructive lung disease, *COPD* Chronic obstructive pulmonary disease, *ICS* Inhaled corticosteroids, *LABA* Long-acting beta2-agonist, *LAMA* Long-acting muscarinic antagonists, *SGRQ* St. George’s respiratory questionnaire, *6MWD* 6 min walking distance, *MBL* Mannose binding lectin, *SABA* Short-acting beta2-agonist, *SAMA* Short-acting muscarinic antagonists, *SF-36* Short form (36) health survey

### Serum MBL levels and association with patient characteristics

The median serum MBL level in our study cohort was (612 ng/ml; [85–2,363 ng/ml]). There was no correlation of serum MBL and FEV_1_% pred. post BD (*p* = 0.36). The median serum MBL in COPD II patients was (590 ng/ml, [IQR; 106–2106 ng/ml]), COPD III (659 ng/ml, [IQR; 94–2,363 ng/ml]), and COPD IV (491 ng/ml, [IQR; 85–2,120 ng/ml]). There was no difference in serum MBL level between frequent and infrequent exacerbators (median, 612 ng/ml vs. 615 ng/ml; *p* = 0.59). MBL level showed no difference between patients who died from COPD (4.33 %) and patients who died due to other reasons (6.13 %) (median, 625 ng/ml vs. 590 ng/ml; *p* = 0.49).

At stable state, 90 patients provided sputum of which 26 yielded positive culture results. The isolated organisms were: *Pseudomonas aeruginosa* (*n* = 10), *Enterobacter spp.* (*n* = 7)*, Streptococcus pneumoniae* (*n* = 6)*, Haemophilus influenzae* (*n* = 6)*, Moraxella catarrhalis* (*n* = 4)*,* and *Staphylococcus aureus* (*n* = 1). Patients with and without evidence of chronic bacterial colonization had comparable circulating MBL levels (754 ng/ml; IQR, 559–1113 ng/ml vs. 627 ng/ml; IQR, 455–915 ng/ml, *p* = 0.30). Serum MBL was similar in colonization with Gram positive vs. Gram negative staining (762 ng/ml, [IQR 559–909 ng/ml] vs. 942 ng/ml, [IQR, 637–1,294 ng/ml], *p* = 0.38).

**Fig. 1:**
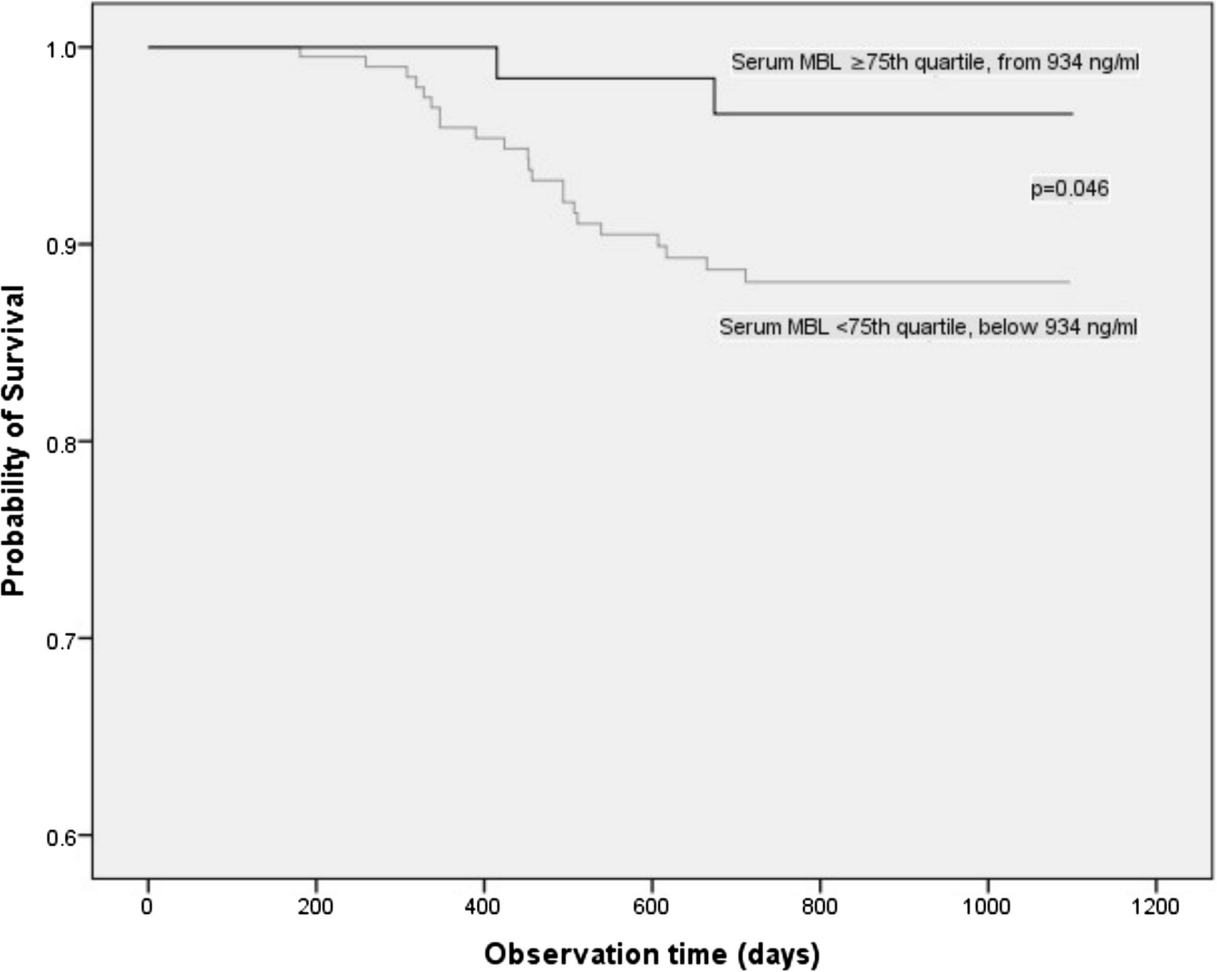
Kaplan-Meier survival analysis of patients with low and high serum MBL levels in COPD patients. The survival analysis of time to death showed significant survival advantage among patients with high serum MBL levels as compared to those with low serum MBL levels (Log rank, *p* = 0.046). MBL = Mannose binding lectin

The distribution of co-morbid state was comparable between frequent and infrequent exacerbators and was independent of serum MBL. A logistic regression analysis was performed to evaluate the serum MBL and its association to patient characteristics including exacerbation rate. Serum MBL was not associated with COPD duration, age, BMI, GOLD stage, post-FEV_1_% predicted, SGRQ total score, arterial hypertension, *MBL2 H/L* allele, *MBL2* exon 1 polymorphisms (*A/A,* vs. *A/O, O/O*) (*p* > 0.05). In the low serum MBL group, 22 patients (10.6 %) deceased during the study period as compared to 2 patients (2.9 %) in the high serum MBL group. The hazard ratio for death within 2 years for patients with high serum MBL was 4.0 (95 % CI, 0.94–50; *p* = 0.05). The survival rate in patients with high serum MBL was higher than in those with low serum MBL (Fig. 1, *p* = 0.046, log rank test).

### Polymorphisms in *MBL2* gene

Analysing the frequency of SNPs in the *MBL2* promoter region (*H/L*), we observed a statistically significant difference with twenty nine (23.6 %) of the 123 frequent exacerbators being *H/H* homozygotes compared to twenty (13.0 %) in 154 infrequent exacerbators (*p* = 0.043). Similarly, the distribution of the *A/D* allele was significantly different in frequent and infrequent exacerbators (*p* = 0.03). However, there was no difference in exacerbation frequency for the other allele (*Y/X, P/Q, A/B, A/C*, *p* > 0.05 for all*)*.

In order to test if *MBL* genotypes of exon 1 alleles are associated with COPD outcomes (exacerbation frequency), we grouped the genotypes into two: where the *MBL2* polymorphisms 52 (*A/D*), 54 (*A/B*), and 57 (*A/C*) alleles can be grouped and referred as genotype “*O*” that is considered to express low serum MBL, while the other alleles were assigned as genotype “*A*” that express normal serum MBL levels [16]. In our patient cohort, the frequency of the “*O*” genotype was not significantly different in infrequent and frequent exacerbators (27.9 % vs. 31.7 %; *p* = 0.47), and, the serum MBL level was independent of exon 1 alleles (*p* = 0.39).

In a second approach, we combined the promoter *X/Y* allele with the pooled exon 1 *A/O* alleles which is considered to change serum MBL level [17]. In our cohort, the *XA/XA* and *YO/YO* genotypes were the least frequent, however the distribution of all possible six genotypes was not different between frequent and infrequent exacerbators (*p* > 0.05, for all), and no association with serum MBL was observed (*p* > 0.05) (Additional file 1: Table S1).

### Frequencies of *MBL2* haplotypes and serum MBL

The *MBL2* haplotype frequency in infrequent and frequent exacerbators is summarized in Table 3. To further investigate the serum MBL and its function, we compared the MBL haplotypes to serum MBL concentration and patient’s characteristics. Haplotypes (*LYPB, HYPD,* and *LYQC*) that code for low serum MBL were less frequent represented by 15.6 % of the cohort. Based on an earlier report we categorized the *MBL2* polymorphisms as: “high”, “intermediate”, and “low” serum MBL concentration expressing genotypes [18–21], and compared it to exacerbation frequency [15]. However, in this cohort neither the exacerbation frequency nor the serum MBL was different across “MBL concentration genotypes”. Similar median values of serum MBL were observed in infrequent exacerbators (614.5 ng/ml; IQR, 371.0–942.0 ng/ml), and frequent exacerbators (607.0 ng/ml; IQR, 363.0–896.0 ng/ml; *p* = 0.91). Patients with the *HYPD* haplotype had similar hospitalization history, risk to congestive heart failure, arterial hypertension, and death rate (*p* > 0.05) compared to all other haplotypes. However, we found significantly more *HYPD* haplotype carriers among the frequent exacerbators (*p* = 0.01) (Table 3).

**Table 3:** Frequency of *MBL2* haplotypes in relation to exacerbations

*MBL2* Haplotype	Expected serum MBL Level	Infrequent exacerbation		Frequent exacerbation		*p* value
		n	Frequency	n	Frequency	
*HYPA*	High	87	0.282	85	0.346	0.13
*LYQA*	High	75	0.243	53	0.215	0.49
*LYPA*	High	30	0.097	15	0.061	0.16
*LXPA*	Intermediate	71	0.230	50	0.203	0.50
*LYPB*	Low	27	0.087	19	0.077	0.77
*HYPD*	Low	10	0.032	21	0.085	0.01
*LYQC*	Low	8	0.026	3	0.012	0.39

The distribution of haplotypes corresponding to express high to low serum MBL are categorized in infrequent and frequent exacerbation. Here, the *HYPD* haplotype is significantly higher among frequent exacerbators

## Discussion

In this study, we present three important new findings. Firstly, we found that significantly more *HYPD* haplotype carriers, supposed to express low serum MBL levels, were frequent exacerbators. Secondly, serum MBL was neither predictive for COPD severity, exacerbation frequency, nor for bacterial colonization. And finally, low serum MBL at stable state was associated with reduced survival. To our knowledge this is the largest and most comprehensive study assessing both serum MBL and *MBL2* haplotypes in Caucasians COPD patients.

It has been argued that COPD exacerbation involves the innate immune response, which is linked to serum MBL, and *MBL2* gene polymorphisms [3, 22]. The *MBL2* genotypes result from several polymorphisms in both the promoter and exon 1 region and are assumed to modify serum MBL levels [15, 23]. The promoter allele *X/Y* was linked with pooled exon 1 (*A/A, A/O, O/O*) polymorphisms to form genotypes (e.g. *YA/YA, XA/YO*), which account for increased serum MBL. It has been suggested that the promoter *X* allele causes low expression of MBL, while any polymorphisms in exon 1 were associated with reduced MBL function in Caucasians [24]. Previous studies showed that the most commonly described *MBL2* haplotypes *(HYPA, LYPA, LXPA, LYQA, HYPD, LYPB, LYQC*) predict susceptibility to various diseases [25–27]. In this COPD cohort we describe for the first time a significant difference for the allele frequency of *H/L* and *A/D* in frequent and infrequent exacerbators.

The high frequency of the *HYPA* (31 %) haplotype was expected, as it is the most common haplotype among Europeans. However, the *LYPB* (8 %) haplotype was less frequent than described elsewhere [28], but was significantly increased in frequent exacerbators. Previous studies found the *HYPD* haplotype to modulate the risk for liver disease in cystic fibrosis [27, 29], while it was protective against pulmonary tuberculosis [25]. Our data proposes the *HYPD* haplotype as a marker of frequent exacerbation in COPD, while it was not linked to serum MBL levels. It is indicated that serum MBL <100 ng/ml present a deficiency and occurs in 5 % Europeans [30]. In contrast, only 2 (0.7 %) patients in our cohort presented serum MBL <100 ng/ml. Since only MBL sufficient genotypes responded to acute phase reactivity in pneumonia, serum MBL may not significantly increase in patients with MBL deficiency genotypes despite inflammation [31].

In cystic fibrosis, MBL2 levels correlated with reduced FEV1 % predicted [32]. Conversely, in COPD patients at stable state, we found only a trend for a correlation of serum MBL and FEV_1_% predicted. Herein, the additional lack of association of serum MBL with other COPD parameters such as quality of life, BODE index, exercise capacity, or duration of symptoms supports the notion that serum MBL does not directly relate to the severity of the disease in COPD. It has been reported earlier that MBL deficiency showed no association to COPD severity [33]. Studies have shown that MBL deficiency may have detrimental consequences on the long-term outcomes in chronic granulomatous disease (CGD) [34] and those with variant forms of MBL have a high risk of developing autoimmune disorders [35].

In other diseases, such as cystic fibrosis, it was reported that MBL2 deficiency was associated to infection with *Pseudomonas aeruginosa* and *Burkholderia cepacia* [32]. However, in our study, circulating MBL levels in patients with and without bacterial colonisation were similar. Specifically, colonization by *Pseudomonas aeruginosa* was not associated with lower circulating MBL levels. Therefore, the association of MBL deficiency and *Pseudomonas aeruginosa* may be disease specific. [32], Regarding the link of MBL alleles and infection susceptibility we found no difference between patients with and without colonization and serum MBL levels, while it was suggested that *Y/Y* promoter homozygotes are more susceptible to bacterial colonization [36]. *MBL2* polymorphisms had been presented to increase the risk of pneumonia and low serum MBL was controversially correlated with an increased risk of bacterial and viral infections [31]. Furthermore, a large study in Caucasian showed no association of exon 1 *MBL2* genotypes and infection [37]. Bacterial surface lipopolysaccharide capsular antigen of *Streptococcus pneumoniae* causes airway inflammation, stimulating and binding with MBL protein and potentially leading to exacerbations [38]. Another study found the high-MBL expressing *HYPA* haplotype more frequent among controls than in patients with infection [39]. In isolates from immune-compromised children, MBL protein bound only to a selection of bacteria [40]. Most of these strong MBL binding bacteria were not encountered in the present COPD cohort suggesting a limited involvement in innate immunity. Yang et al. described a significant association between the *MBL2*-54 *A/B* allele and reduced serum MBL with infective COPD exacerbation. Unfortunately, exacerbations were not further differentiated for microorganisms [41]. In the present cohort, we observed a better survival in patients with high MBL levels. In accordance with our findings, Dahl et al. reported a higher prevalence of cardiovascular disorders in deficient *MBL2* carrier patients [37]. In kidney transplant patients, the low serum MBL haplotypes *XA* and *YO* were also significantly associated with reduced survival [42]. These results are in agreement with a study in the United Kingdom showing that lower circulating MBL level was related to poor disease outcome in septic shock [43]. Although this study lacks the ability to define precise mechanisms of the role of serum MBL to disease pathogenesis, our findings suggest that high serum MBL is protective in COPD.

We have observed that increased MBL levels correlated with increased survival, Shall its protective effect be confirmed in further studies, it would be worthwhile exploring recombinant or purified MBL replacement as a therapeutic strategy in high-risk patients with COPD.

### Limitations

The limitations of this study are: we did not have a control group, which would have added value to this analysis. However, the distribution of *MBL2* polymorphisms in our population was similar to that reported in the dbSNP database [44]. We cannot exclude the possibility that an association between deficient MBL2 genotypes and serum MBL concentrations could be evidenced in a population with a different ethnic background and/or larger sample size. There is no consensus in defining deficient serum MBL levels, thus we used the 4^th^ quartile as threshold value. As any data driven definition, this threshold might overestimate any association. Taking in to account that MBL might exert a local effect in the lung, which is not reflected in the serum, it would be of interest to compare serum to BAL samples. However in the setting of this study BAL samples were not collected. The range of MBL in BAL samples is at least 100 times greater than what we observed in serum samples (ng/ml vs pg/ml). Therefore the variation in our samples is significantly smaller as compared to that of Hodge et al. [45]. It would be of interest to determine the influence of MASP2 with MBL2 as only the complex of both proteins exerts an anti-bacterial/viral effect. However, to include this data in our cohort we would also have to determine all known polymorphisms for MASP2 and then combine them with all genotypes of MBL2. Similarly, the association between HYPD haplotype and frequent exacerbations should be confirmed in further studies. Nevertheless, the major strength of this study is the detailed patient and disease activity characterisation of a pan-European COPD cohort.

## Conclusions

The major findings of this study suggests that Caucasian COPD patients with the *MBL2-HYPD* haplotype demonstrate more commonly frequent exacerbations, while high serum MBL at stable state is associated with increased survival.

## Additional file

Additional file 1: Table S1.
**Distribution of serum MBL level and exacerbation frequency in different "MBL concentration groups".** (DOCX 89 kb)

## Abbreviations

BD: broncho-dilator
BODE: body mass index, airflow obstruction, dyspnea and exercise capacity
COPD: chronic obstructive pulmonary disease
ELISA: enzyme linked immunosorbent assay
FEV_1_: forced expiratory volume one second
FVC: forced vital capacity
GOLD: global initiative for chronic obstructive lung disease
IQR: interquartile range
MASP: MBL associated serine protease
MBL: mannose binding lectin
PROMISE-COPD: predicting outcome using systemic markers in severe exacerbations of COPD
RT-PCR: real time- polymerase chain reaction
SF-36: short form (36) health survey
SGRQ: St. George’s respiratory questionnaire
SPSS: statistical package for social sciences
