# Supplementary material for: Mannose-binding lectin protein and its association to clinical outcomes in COPD: a longitudinal study
Source: Respir Res. 2015 Dec 18;16:150. doi: 10.1186/s12931-015-0306-3 (PMC4750539; doi:10.1186/s12931-015-0306-3)
Supplement: Additional file 1: Table S1. — Distribution of serum MBL level and exacerbation frequency in different "MBL concentration groups". (DOCX 89 kb) [file 12931_2015_306_MOESM1_ESM.docx]

(Supplementary table 1)

Distribution of serum MBL level and exacerbation frequency in different "MBL concentration groups"

| MBL concentration group* | Genotype | Infrequent exacerbation,  N (%) | Frequent exacerbation,  N (%) | MBL median value in Infrequent exacerbators  ng/ml; Median (IQR) | MBL median level in Frequent exacerbators  ng/ml; Median (IQR) |
| --- | --- | --- | --- | --- | --- |
| High | *YA/YA* | 49 (36.3) | 59 (41.5) | 610 (130-1736) | 607 (85 – 2363) |
|  | *YA/XA* | 38 (28.1) | 33 (23.2) | 624 (115 – 1875) | 637 (117 – 2120) |
|  | Subtotal | 87 | 92 |  |  |
| Intermediate | *XA/XA* | 9 (6) | 7 (4) | 364 (264 – 1749) | 700 (308 – 1304) |
|  | *YA/YO* | 27 (20) | 28 (19.7) | 611 (94 – 1471) | 572 (106 – 2092) |
|  | Subtotal | 36 | 35 |  |  |
| Low | *XA/YO* | 10 (7) | 8 (5) | 524 (108 – 1458) | 573 (101 – 1069) |
|  | *YO/YO* | 2 (1.5) | 7 (4.9) | 909 (534 – 1283) | 824 (231 – 956) |
|  | Subtotal | 12 | 15 |  |  |
| Both the genotype frequency and the median MBL level were not significantly different in infrequent and frequent exacerbators (p>0.05).  References   1. Olesen HV, Jensenius JC, Steffensen R, Thiel S, Schiotz PO: The mannan-binding lectin pathway and lung disease in cystic fibrosis--disfunction of mannan-binding lectin-associated serine protease 2 (MASP-2) may be a major modifier. Clin Immunol 2006, 121**:**324-31. 2. Chalmers JD, McHugh BJ, Doherty C, Smith MP, Govan JR, Kilpatrick DC, et al: Mannose-binding lectin deficiency and disease severity in non-cystic fibrosis bronchiectasis: a prospective study. Lancet Respir Med 2013, 1**:**224-32. | | | | | |
